# The Report of Bethlehem Hospital

**Published:** 1853-10-01

**Authors:** 


### THE REPORT OF BETHLEHEM HOSPITAL.

The annual report of this hospital for the year 1852, addressed to its president, treasurer, and governors, will be received with unusual interest; inasmuch as it may reasonably be expected to contain some indication of the future policy which will be adopted in the administration of this great but notoriously mismanaged public charity. No institution founded for the reception, care, and treatment of the insane is more ancient than Bethlehem; and none can record darker passages in its history. The scenes which once excited the curious wonderment of Pepys, Hogarth, Fielding, Johnson—and which have been depicted with graphic minuteness by the literary artists of a by-gone age, humanity now recoils from; we cannot be surprised, therefore, at the public indignation which was expressed when the commissioners in lunacy made known the results of their recent investigation. We had no idea that such abuses, as they described, existed now-a-days in any lunatic asylum—at all events, in this kingdom; but regrets are now vain. The secrets of the prison-house have been revealed, and we have reason to believe that the Augean stable has been thoroughly cleansed. Immediately after the commissioners' report was published, a sudden change—a complete reformation—took place in every department of the establishment. The consulting physicians resigned. The resident medical officer followed their example—the matron withdrew—many of the attendants, male and female, were discharged; and the governors, shaking off the lethargy which often encumbers the sceptre of authority, have, we are informed, been since unceasingly on the alert. Their vigilance is now almost miraculous; some of them are always to be seen moving about the building; many it is supposed never sleep; but, be this as it may, for we will not vouch for the truth of apocryphal jests, this much is certain, that the Governors of Bethlehem meet more frequently in committee than her Majesty's Ministers in Council; and that they are making strenuous efforts to uncloud and vindicate the character of an institution which ought to enjoy pre-eminence among the most useful and important charities in Europe. The officers to whom we have alluded having withdrawn, their successors were soon appointed; and we shall not upon this occasion reopen the discussion respecting the propriety of their elections. They have now entered upon their several duties,—*fiat justitia* be our motto—we desire that they may have fair play, and shall content ourselves with hoping that the new system (which we may observe is scarcely yet organized) will be found to work well, and that a brighter era will ere long dispel the gloom which obscures the immediate vista of the past.

The report which Dr. Hood has laid before us is modest and unassuming; he does not congratulate himself upon his appointment, or indulge in any Io-pæans on the occasion; he makes no high-sounding promises of what he proposes to do, but addresses himself, without any flourish of trumpets, to the immediate business—the subject-matter of the report. It states that, having so recently entered upon the duties of his office, he has not yet had time to mature his observations on many subjects connected with his department to which he should otherwise have called our attention; and that he must of necessity found his present report upon such information as he finds upon the records of the institution. He therefore abstains from referring to cases which were under the charge of his predecessors, fully detailed as they may be in the books of the hospital, and avails himself of the tabular forms which have been hitherto adopted to exhibit the usual statistical details of admissions and discharges, recoveries and deaths, and other details elaborately given in these tables, which, if we remember correctly, were devised by the able and indefatigable Dr. Webster, who, as a governor, has never spared his professional services in the midst of his official duties. We conceive this to be, on the part of Dr. Hood, in good taste. It would have been indelicate for him to have commented upon the cases under treatment by his predecessors; and the tabular forms referred to are extremely simple, lucid, and complete; presenting, under appropriate columns, all the statistical details that can be desired. During the year 1852, there appear to have been 303 patients admitted into Bethlehem, and 252 discharged, of whom 145 were cured, and 107 uncured. We are indebted mainly, we presume, to the researches of Dr. Webster for the following return of the total number of curable patients admitted into Bethlehem Hospital during ONE HUNDRED years ending 31st December, 1852, with the amount of cures and deaths:—

| | | |
|---|---|---|
| Total patients admitted . . | 19,192 | |
| Discharged cured . . . . | 8086 . . | or 42·13 per cent. |
| Died . . . . . . . . . | 1675 . . | or 8·72 per cent. |

All the tables in the Report before us are exceedingly curious and valuable —but must be compared with the returns of other asylums, before any legitimate deductions can be drawn from them. On the 31st of December, 1852, there were in Bethlehem 351 inmates, of whom 193 were males, and 158 females, and these were disposed as follows:—

| | M. | F. | TOTAL. |
|---|---|---|---|
| Curable . . | 65 | 99 | 164 |
| Incurable . . | 38 | 37 | 75 |
| Criminals . . | 90 | 22 | 112 |
| | 193 | 158 | 351 |

Of the 112 criminal lunatics, 90 were male and 22 female. The following tables will show the offences which were committed by these persons, and the time they have been in Bethlehem:—

*Synopsis of Offences of the Criminal Lunatics confined in Bethlehem Hospital,* 31*st December*, 1852.

| NATURE OF OFFENCES. | MALES. | FEMALES. | TOTAL. |
|---|---|---|---|
| 1. Against the State. | | | |
| (1.) High Treason . 1 | | | |
| (2.) Sedition . . . 1 | 2 | .. | 2 |
| 2. Against the Person . . | 66 | 19 | 85 |
| 3. Against Property . . . | 17 | 3 | 20 |
| 4. Other Offences . . . . | 5 | .. | 5 |
| | 90 | 22 | 112 |

*Time the Criminal Patients have been in Bethlehem Hospital.*

| | MALES. | FEMALES. | TOTAL. |
|---|---|---|---|
| 35 years . . . . . . . . | 1 | 1 | 2 |
| 30 " . . . . . . . . | 4 | 1 | 5 |
| 25 " . . . . . . . . | 3 | 2 | 5 |
| 20 " . . . . . . . . | 2 | 1 | 3 |
| 15 " . . . . . . . . | 2 | .. | 2 |
| 10 " . . . . . . . . | 12 | 3 | 15 |
| 5 " . . . . . . . . | 13 | 3 | 16 |
| 3 " . . . . . . . . | 11 | 2 | 13 |
| 2 " . . . . . . . . | 7 | 5 | 12 |
| 1 " . . . . . . . . | 11 | 1 | 12 |
| Admitted during the year 1852 . | 24 | 3 | 27 |
| | 90 | 22 | 112 |

The most interesting portion of Dr. Hood's report, and that which most immediately concerns the public, and the well-being of the charity, consists in the improvements which are stated to be in progress. The following is a summary of them. The number of the attendants has been increased, with a higher amount of wages, with the view of securing the services of persons who are undoubtedly respectable as well as humane and intelligent. A head nurse has been appointed to the female side of the hospital, and an officer of similar rank is placed on the male side. The rules for the guidance of the matron have been revised; and she is under orders to visit frequently, both day and night, the female wards. A more comprehensive form of ward report has been adopted, which will record the principal incidents that occur. The appointments connected with the sleeping-apartments of the patients have also been improved, upon which subject Dr. Hood very justly observes,—"I am convinced that attention to the most minute particulars in their apartments—the appearance of neatness, cleanliness, and home-like comforts, has a soothing and salutary effect upon the mind of all insane persons. Even though unaccustomed to habits which prevail among the wealthier classes, the humblest artisan quickly appreciates any improvement in his social condition; as soon as he acquires a conscious feeling of self-respect, and the *morale* of his being becomes, as it were, raised within him, a curative process is mentally established which rapidly conduces to his recovery. It is of the greatest importance, therefore, to surround the insane with all those conditions which may contribute to their personal accommodation, whereby thoughts and associations of a healthful tone and character may be suggested, which will gradually dispel the delusions that afflict them."—(p. 47.) All this is well and happily expressed; and it is satisfactory to hear that the massive iron bars which now disfigure and give a prison-like aspect to the windows of this magnificent edifice (no finer building, in point of architectural beauty and majesty, exists in London) are to be removed, and replaced by windows of a lighter and more graceful construction. "Experience," adds Dr. Hood, truly, "has sufficiently proved the great importance of abolishing every trace of those unhappy circumstances traditionally connected with lunatic asylums. Every outward sign of restraint should be dispensed with, while even the very appearance of personal liberty being circumscribed should be avoided as much as possible, and the vigilance of attendants substituted for heavy prison walls and revolting instruments of torture." —(p. 48.) Additional rooms for the sick and the infirm are also being erected, which, we are assured, will be lofty, spacious, and replete with every convenience; in addition to which, arrangements are in progress to improve and perfect the system of classification. All this is as it should be; and clearly enough indicates an activity in improving—we might almost say in remodelling —the arrangements of this establishment, of which it stood obviously very much in need. We hope, therefore, that the good work which has been commenced will be proceeded with liberally; and we shall look with much interest for Dr. Hood's next Annual Report. We have a pretty good idea of the difficulties by which he is surrounded, but he must be firm in maintaining a position, which entails upon him the responsibility of insisting upon these and all other improvements which can in any way ameliorate the condition of the insane who are now under his charge being efficiently carried out. We cannot conclude these observations without warmly commending the following observations by Dr. Hood, which we are sure will be well received by the profession. "The facts which occur, and the information which may be obtained in this institution, ought not, I conceive," says Dr. Hood, "to be confined within its walls; it is for the interest of science and humanity that they should be made known; and I shall be ever ready to communicate any observations which may appear to be important, to my medical brethren, with the view of extending to them, as far as the powers intrusted to me will permit, the advantages to be derived from this noble charity."

